# Toward Sustainable Healthcare Systems: A Low and Middle-Income Country’s Case for Investing in Healthcare Reforms

**DOI:** 10.7759/cureus.39345

**Published:** 2023-05-22

**Authors:** Almas F Khattak, Aziz Ur Rahman, Madiha Khattak, Mustafa Qazi, Humaira Gilani, Arsalan Khan

**Affiliations:** 1 Community Medicine and Research, Northwest School of Medicine, Peshawar, PAK; 2 Urology, Northwest General Hospital and Research Center, Peshawar, PAK; 3 Physiology, Khyber Medical College, Peshawar, PAK; 4 Medicine and Surgery, Northwest School of Medicine, Peshawar, PAK; 5 Dermatology, Northwest General Hospital and Research Center, Peshawar, PAK

**Keywords:** low and middle-income country, governance, policy, sustainable, sustainable development goals, healthcare service delivery, financing, global healthcare systems, health system reform, healthcare

## Abstract

Pakistan, a low and middle-income country (LMIC), faces challenges in providing sustainable health care to its population due to inadequate financing, weak healthcare infrastructure, and insufficient health human resources. These challenges are not unique to Pakistan and are faced by many LMICs globally. In this paper, we aim to identify key strategies for achieving sustainable healthcare systems in Pakistan and to draw lessons for LMICs globally, keeping in view the healthcare reforms in Pakistan.

We conducted a comprehensive literature review of existing policies and practices related to healthcare financing, service delivery, health information and communication technologies (ICTs), governance and leadership, and health human resources in Pakistan and other LMICs. We also reviewed relevant global policies and frameworks, including the Sustainable Development Goals (SDGs) and the World Health Organization's (WHO) health system strengthening guidelines.

To achieve sustainable healthcare systems in Pakistan, we recommend comprehensive healthcare financing policies, including increasing budgetary allocations for health, social health protection through universal coverage, and supporting health and economic development processes. Service delivery can be improved through restructuring public health facilities, incorporating behavioral and social health determinants into primary health care, aligning healthcare delivery with the community, and promoting collaborative leadership between the public and private sectors. The use of ICT can be expanded by implementing e-health policies, disseminating authentic public health information, and enabling telemedicine services. Effective healthcare governance and leadership can be promoted through meritorious, transparent, and accountable reforms, stable healthcare structures at all community levels, and appropriate health policy and organizational frameworks. Finally, strengthening health human resources can be achieved through compliant policy implementation and revisions in laws and policies governing medical teaching institutions.

Achieving sustainable healthcare systems in Pakistan and LMICs globally requires comprehensive strategies for healthcare financing, service delivery, health ICT, governance and leadership, and health human resources. By drawing on global policies and frameworks and lessons from other LMICs, Pakistan can overcome its healthcare challenges and contribute to the achievement of the SDGs.

## Introduction and background

The Constitution of 1973 established Pakistan as a federation with a parliamentary system of governance consisting of four provincial units and three administrative territories. In 2018, the Federally Administered Tribal Areas (FATA) were merged into the provincial government of Khyber Pakhtunkhwa (KP) [1]. The constitution laid down two lists of responsibilities: list 1, which includes domains such as military and defense and foreign policy, is the prime responsibility of the federal government, while list 2, which includes domains such as rural development, local government, and health education, is the responsibility of respective provincial governments. Health was included in a third list, the concurrent list of responsibilities, which included subjects jointly overseen by both the central and provincial governments. However, following the implementation of the 18th Amendment in 2010, health became decentralized and the prime responsibility of provincial governments to be managed by their respective health departments [1].

The politically driven decentralization process aimed to empower provincial and district-level policymakers and service providers to make them more accountable to the communities they serve and improve the quality of healthcare services in KP [2,3]. However, how this process has impacted the healthcare system of the province, and the health of its people, remains a topic of ongoing debate.

This paper presents a systematic situational analysis of the healthcare system of a province in Pakistan (i.e., Khyber Pakhtunkhwa) and looks into how the health sector reforms have influenced the healthcare system of the province post-18th Amendment. The paper conducts a cost-benefit analysis of the reforms in terms of healthcare leadership and governance, healthcare service delivery, and health system finance and expenditure, and also evaluates the impact of the reforms on the healthcare workforce and the health system information, communication, and technological advancements in the province. The study aims to provide deeper insight into the implementation hurdles and possible solutions for continuing efficient healthcare service delivery and governance of the province.

The paper presents the profile of the healthcare system in KP, including a background of health system reforms in the province and reform initiatives in the past eight years. It also presents a comprehensive cost-benefit analysis of the provincial health sector reforms in terms of financing and expenditure, health service delivery, leadership and governance, health system information and technology, and the challenges faced so far. The paper concludes with a comparative analysis of other low and middle-income countries (LMICs), recommendations for improving the healthcare system in KP, and a way forward in the global healthcare context.

## Review

Health system profile of Khyber Pakhtunkhwa

The healthcare system in KP has undergone significant reforms in recent years, following the devolution of health as a provincial subject after the 18th Constitutional Amendment. The federal government is responsible for coordination, accreditation, research training, and drug control, while provincial and district governments are tasked with delivering and managing health services [4].

The Department of Health is based in the capital, Peshawar, and includes the Secretariat of Health, Directorate General Health Services, and Provincial Health Services Academy. The department manages various schools, hospitals, and regulatory bodies, including the Health Care Commission, Health Foundation, Directorate General of Medicines and Pharmacy Service, and Medical Transplant Regulatory Authority. Furthermore, 11 autonomous teaching hospitals in the public sector operate with their own generated funds, managed by a board of governors. The healthcare system in KP faces challenges that require ongoing attention and improvement.

The private sector has a significant role in the provision of healthcare services in the KP province of Pakistan. Private healthcare providers offer a wide range of services, including diagnostic tests, outpatient and inpatient care, and specialist consultations. Private hospitals and clinics are more readily available in urban areas, while rural areas rely mostly on public healthcare facilities.

The private sector in KP is regulated by the government to ensure quality healthcare delivery and to prevent unethical practices. However, there are still concerns about the affordability and accessibility of private healthcare, particularly for the poor and vulnerable populations.

Situational analysis

Healthcare delivery in Pakistan was a joint responsibility of federal and provincial governments and was implemented through districts before the process of decentralization. Post-18th Amendment, health care is now the prime mandate of provincial governments. In KP, health services are delivered through preventive, promotive, curative, and rehabilitative services at primary, secondary, and tertiary healthcare facilities along with integrative national programs. The health service delivery system in KP mainly comprises the public and private sectors. Post-devolution, the health workforce has considerably increased to provide adequate and effective health services to all communities. Although improvement has been evident, private health facilities are playing an efficient role in bridging the gap of increasing utilization of overburdened public health facilities. Good leadership and governance play a paramount role in delivering quality healthcare services to the public through a transparent and accountable healthcare delivery system.

This section evaluates the policymaking situation of medical teaching institutions (MTIs) in KP, and the costs incurred on the healthcare system in terms of both tangible and intangible costs (monetary, material, and human costs) for the following key domains of the healthcare system in KP post-18th Amendment.

Legal Framework

The Khyber Pakhtunkhwa Medical Teaching Institutions Reforms Act of 2015 grants autonomy to government-owned MTIs and their affiliated teaching hospitals in the province of KP to improve their performance, effectiveness, efficiency, and responsiveness in providing quality healthcare services. The act has been implemented in eight hospitals and mandates effective monitoring and periodic quarterly evaluation of their performance [5].

MTIs Situation in KP

Despite large investments in health infrastructure, the lack of proper resource utilization, rather than the lack of resources, is the problem. The government spends almost 50% of provincial health expenditures on hospitals [6], yet MTIs suffer from serious quality problems, inefficiency, inappropriate financial management and information systems, low staff morale, and staff absenteeism. Bed occupancy rate (BOR) and type of service provision are not commensurate with the status of tertiary care hospitals. The public sector-private sector dichotomy is another issue.

Health Sector Expenditures

An analysis shows that health sector expenditures in KP are four times higher in the private sector than in the public sector [7], and KP has the highest share of out-of-pocket expenditure for health care. The incidence of catastrophic health expenditures is more in KP [8]. The MTIs have the flexibility to develop their vision, mission, strategic direction, and organizational culture under the autonomy granted to them by the MTI Act 2015, but they have not been able to use it to their full potential [9]. Therefore, there is a need for hospital reforms to improve management capacity, staff motivation, accountability, willingness to innovate, and emphasis on measurement of results.

Potential Benefits to the Provincial Health System

This paper analyzed the benefits of the 18th Amendment to the provincial health system in four domains. Firstly, the devolution of powers to provinces has led to greater autonomy and political ownership of health at the provincial and district levels, as evidenced by the health sector reforms in KP. Secondly, the improvement of primary health has become a priority, and different provinces have taken steps to improve it. KP is ahead of Punjab and Sindh in terms of planning, service delivery, and contracting with the private sector. Thirdly, the provincial government's efforts to improve healthcare services are congruent with the national health policy of Pakistan, which aims to provide equitable healthcare services and universal access to health care. Finally, the government's commitment to the global slogan of "Health for All" is evidenced by its efforts to make health care accessible to all through Sehat Insaf Card [10]. Overall, the devolution of powers to provinces has led to increased allocation of resources, good budgetary use, and pragmatic planning of the provincial government to improve the overall healthcare system of KP [11].

Assessing Health System Performance in KP: Examining Patient Experiences and Population Health Outcomes

The paper evaluated the health system performance of KP in terms of outcome measures. Outcome measures include mortality, readmission, and patient experience, which are the quality and cost targets healthcare organizations aim to improve. Two outcome measures were evaluated: improvement in patient experiences of healthcare services and improvement in population health (morbidity and mortality indicators data).

To assess the improvement in patient experiences, the utilization of services in 2019 was compared to that of the same services in 2018. The results showed an increase in normal deliveries at primary and secondary healthcare facilities and cesarean sections. Table 1 shows the comparison of services provided in 2018 and 2019 [12].

**Table 1:** Comparison of services provided to people Source: Government of Khyber Pakhtunkhwa, Health Department 2019.

	2018	2019	Increase
OPD at secondary hospitals	15,961,607	16,328,996	2%
Emergency cases	4,244,150	4,367,877	3%
Normal delivery at a primary facility	36,959	40,617	10%
Normal delivery at a secondary facility	145,741	171,717	18%
Cesarean section	15,659	20,520	31%
Diagnostic services	6,758,986	6,994,070	3%
Surgeries	203,136	228,502	12%

However, despite the provincial government's efforts, KP's performance in determining key health outcome measures remains poor. Maternal health shows an extremely high maternal mortality rate, and child health shows a dangerously high infant mortality rate, with alarming stunting and wasting among children. Immunization coverage is also low. In addition, the situation in secondary care is poor, with a low number of doctors and beds per 10,000 population [13].

Skilled birth attendance has increased, but there are still many deliveries occurring at home, and not all children are fully vaccinated. Key indicators, such as maternal mortality rate, child health, immunization, and malnutrition, continue to hamper progress in the health sector. There is also an alarming increase in non-communicable diseases and the aging population, which requires a revision in health policy and services.

Challenges in implementing health services post-18th Amendment

Unclear Roles and Responsibilities of Different Tiers

The lack of clear-cut roles and responsibilities at district, provincial, and federal levels in health service delivery has created confusion and challenges in proper implementation post-devolution.

Inadequate Financing for Health Services at Provincial Level

Post-amendment, the fiscal transfer financing at the provincial level has not been re-examined, and the federal government's funding for social services and infrastructure is insufficient to set national standards for health expenses.

Organizational Fragmentation and Delayed Fund Release

The central government's poor organizational structure in implementing the major functions of the amendment has led to fragmented service delivery, duplication of resources, and delayed fund release for vertical health programs due to budget retention.

Regulatory Challenges and Confusion

The devolution of powers to the provinces has created potential confusion and contradictions regarding standards and administration costs in pharmaceutical regulations, food and agricultural safety, hazardous material control and disposal, waste, water and air pollution, highway safety, consumer product safety, and social safety nets. The lack of an effective regulatory mechanism adds to the challenge.

Lack of Transparency and Accountability in Decision-Making

The politically motivated accountability systems have kept citizens estranged from decision-making processes, hampering transparency and accountability.

Administrative Inefficiencies and Limited Participation

The bureaucratic and executive level powers with extensive provincial control have created issues and aggravated inefficiencies in providing smooth public health services. Limited interaction and faulty check and balance mechanisms with district administrations have also limited private and non-profit organizations' participation in improving public health service access.

Health reforms in other countries: lessons learned

Sustained fundamental changes in policies and healthcare institutions ultimately culminate into impactful reforms in the health sector. Good health sector reforms are based on equity, efficiency, quality, financing, and sustainability, with well-defined priorities, policies, and reformed institutions to implement them in true essence. Post-18th Amendment and specifically in the last seven to eight years, the new political powers in KP realized the gravity of challenges and the opportunities and potential for further improvement in health sector development to ensure access to health care for all. Since then, the government has been striving hard to mitigate issues such as reducing inequities in health, self-reliance in resources, provision of basic health services to all, and maintaining health ethics. Therefore, we provided an overview of health system reforms in other countries and compared them to the changes that have been initiated in Pakistan, particularly in the province of KP after the decentralization process post-18th Amendment.

China, Vietnam, and Mexico vs. Pakistan

China's New Cooperative Medical Scheme, Vietnam's Health Care Fund for the Poor, and Mexico's System for Social Protection in Health, all promote universal access to health care, provide quality health services, and ensure fair financing and expenditures on health. Pakistan's Sehat Sahulat Program (SSP) is a pragmatic step toward social welfare reforms in the province to ensure that underprivileged citizens receive entitled medical care in an easy and dignified manner without having to worry about enormous health expenditures.

Pakistan vs. Bangladesh

Both Pakistan and Bangladesh have struggled and are still struggling to improve their healthcare delivery systems. While Pakistan has better and vast infrastructural facilities, Bangladesh has made significant improvements in its primary healthcare system [14].

India vs. Pakistan

In the 1990s, the health sector in India underwent a significant transformation, which led to the growth of private health care, a cost recovery system in the public sector, and an efficient and socially accountable public healthcare system. These reforms aimed to promote health equity, encourage public-private partnerships in healthcare expenditure, improve the quality of treatment, and address the factors affecting the delivery of healthcare services. However, like other developing countries, India's healthcare expenses are primarily out of pocket, which disproportionately affects the health of those living in absolute poverty. In comparison to Pakistan, healthcare research and technological advancements are given higher priority in India. This comparison is important because both countries share similar socioeconomic dynamics [15,16].

Cuba

Cuba has a publicly funded and universally accessible healthcare system, which has been praised for its focus on preventive care, early detection and treatment of diseases, and low-cost medicines. Despite facing economic challenges and limited resources, Cuba has achieved impressive health outcomes, with life expectancy and infant mortality rates that are comparable to those of developed countries.

Sri Lanka

Sri Lanka has a free healthcare system that is accessible to all citizens, regardless of their income or social status. The government has invested heavily in the healthcare sector, with a focus on improving access to essential medicines, reducing maternal and child mortality rates, and controlling communicable diseases. As a result, Sri Lanka has achieved impressive health outcomes, including one of the lowest maternal mortality rates in South Asia [17].

Thailand

Thailand has a universal healthcare system that provides access to quality healthcare services to all citizens. The system is funded by the government through taxes and has been successful in achieving universal health coverage [18]. Thailand has also invested heavily in research and development in the healthcare sector, particularly in the areas of tropical medicine and infectious diseases.

United Kingdom

The United Kingdom has a publicly funded healthcare system called the National Health Service (NHS), which provides free healthcare services to all citizens. The NHS is one of the largest employers in the world [19] and has been praised for its commitment to providing quality healthcare services to all citizens, regardless of their ability to pay. The UK has also invested heavily in medical research and innovation, particularly in the fields of genomics and personalized medicine.

Recommendations

Based on the analysis and discussion presented in this research paper, the following recommendations are suggested.

Increase Investment in Health Care

The government of Pakistan needs to allocate a higher proportion of its budget toward health care to ensure that every citizen has access to basic healthcare services.

Improve Healthcare Infrastructure

There is a need to increase the number of healthcare facilities and improve the existing infrastructure in Pakistan. The government should collaborate with private healthcare providers to establish more hospitals and clinics in rural and remote areas.

Strengthen Primary Healthcare

Primary healthcare services should be strengthened and prioritized, as they provide the foundation for a well-functioning healthcare system. This can be achieved by investing in the training and development of primary healthcare providers and improving access to essential medicines and vaccines by involving stakeholders from the government, academia, and non-governmental organizations and by strong partnerships across all levels of the healthcare system, organizations, and disease areas.

Increase Health Workforce

The government should invest in recruiting and training more healthcare professionals to address the current shortage of healthcare workers in Pakistan. This can be achieved by offering incentives such as time off, regular pay increases, pensions and housing allowances, or by adopting the P4P (payment for performance) program and improving working conditions for healthcare professionals.

Utilize Technology

Technology can play a vital role in improving healthcare delivery in Pakistan. The government should invest in telemedicine and other digital health solutions to increase access to healthcare services in remote areas and improve efficiency in healthcare delivery.

Emphasize Preventive Health Care

There is a need to shift the focus toward preventive health care in Pakistan. The government should invest in public health campaigns to raise awareness about healthy lifestyles, disease prevention, and the importance of regular check-ups.

Ensure Accountability

The government should ensure accountability in the healthcare system by implementing transparent and effective monitoring systems. This can be achieved by establishing an independent regulatory body to monitor healthcare providers and ensure that they adhere to quality standards and ethical practices.

By implementing these recommendations, Pakistan can move toward a sustainable healthcare system that provides universal access to quality healthcare services and improves the overall health and well-being of its citizens.

Way forward

Formulate comprehensive and stable healthcare finance policies at the national level with technical support and training from the WHO.

Increase spending on health by increasing budgetary allocations for health by at least 3-4% of gross domestic product by 2025, with expert solutions for inadequacies in funding, equitable use of resources, capacity building of administrators, and mechanisms for the sustainability of financial management.

Implement social health protection with universal coverage through affordable public and private health insurance schemes for essential health services for all citizens.

Support health and economic development processes through public awareness, coordination between national agencies, collaborations with donor agencies, and accountability.

Strengthen regulatory mechanisms, improve evidence for policy development and implementation, and implement strong target-oriented monitoring and evaluation systems.

Restructure all public health facilities, improve primary health care by incorporating behavioral health and social health determinants in the system, and create highly safe and reliable healthcare organizations by incorporating national public health programs at the district and provincial levels.

Align healthcare delivery and the community by bridging gaps in service delivery and promoting collaborative leadership with a proper system of publicly funded services in partnership with the private sector.

Develop a policy on e-health with periodic analysis, facilitate dissemination of authentic public health information, implement electronic health records at all provincial and district health facilities, enable effective telemedicine services, and develop strong ICT systems to facilitate collaboration and cooperation among health workers, researchers, expert trainers, and policymakers.

Promote meritorious, transparent, and accountable healthcare governance and leadership structures at all community levels, implement appropriate health policy, laws, and organizational frameworks at both federal and provincial levels for stronger health system performance, regulate the private health sector through policies and services packages, and implement clinical and corporate governance to make healthcare organizations accountable to stakeholders and communities.

Develop and implement a health workforce-compliant policy and introduce amendments/revisions in laws/policies governing MTIs with a workable regulatory framework and monitoring and evaluation system in place and appoint an overall administrator/chief executive/chief operating officer to oversee the management, facilitation, and dispute resolution among the directors.

## Conclusions

In conclusion, Pakistan's healthcare system is facing numerous challenges that require immediate attention and action. The country has made some progress in recent years by implementing various health reforms, including the establishment of universal health coverage schemes and investing in the health workforce. However, there is still a long way to go in terms of achieving sustainable healthcare systems that provide accessible, affordable, and high-quality healthcare services to all.

Investing in health reforms that place the needs of the populace first would be essential for Pakistan's healthcare system to succeed in overcoming its difficulties. This entails resolving the scarcity of healthcare workers, enhancing the healthcare system, and expanding access to necessary drugs and vaccines. Achieving sustainable healthcare systems can also be aided by guaranteeing appropriate funding for health care, encouraging public-private collaborations, and utilizing technology to enhance healthcare delivery. Additionally, to address the problems with Pakistan's healthcare system and make sure that healthcare services are available and affordable for everyone, it is crucial for stakeholders and policymakers to collaborate. By investing in health reforms and adopting evidence-based policies, Pakistan can move toward achieving sustainable healthcare systems that improve the health and well-being of its population and contribute to the overall development of the country.
